# Optimizing contrast-enhanced echocardiography by employing a sonographer driven protocol

**DOI:** 10.1007/s12574-021-00523-y

**Published:** 2021-04-02

**Authors:** Courtney R. Usry, Satoshi R. Shin, James K. Aden, Rosco Gore

**Affiliations:** 1 Cardiology Service, William Beaumont Army Medical Center, 5005 N. Piedras Street, El Paso, TX 79920 USA; 2 Division of Cardiology, Brooke Army Medical Center, San Antonio, TX 78234 USA; 3 Research Statistician, Brooke Army Medical Center, San Antonio, TX 78234 USA; 4 Cardiology Department, Naval Medical Center, San Diego, CA 92134 USA

**Keywords:** Contrast echocardiography, Laboratory protocols, Left ventricular function, Sonographer

## Abstract

**Background:**

The use of enhancing agents in echocardiography has been shown to facilitate improved study quality. Despite the known benefits, its use remains limited by institutional policies.

**Methods:**

We aimed to retrospectively evaluate if allowing sonographers to place a peripheral intravenous catheter and administer enhancing agent led to a decrease in time to complete outpatient transthoracic echocardiograms in comparison to using nursing personnel. Three separate protocols were employed. The ‘nurse driven protocol’ utilized nurses to place a peripheral intravenous catheter and inject enhancing agent. In a ‘mixed protocol,’ a nurse placed a peripheral intravenous catheter and the sonographer gave the enhancing agent. The ‘sonographer driven protocol’ involved the sonographer placing the peripheral intravenous catheter and delivering enhancing agent.

**Results:**

A total of 232 echocardiograms were included for analysis. Patient characteristics across the three protocols were not statistically significant. The ‘mixed protocol’ had an average study time that was significantly less than the ‘nurse driven protocol’ (49.4 min ± 11.4 vs 54.6 min ± 12.9; p = 0.024). The ‘sonographer driven protocol’ also showed a significant reduction in study time (50.3 min ± 12.6) when compared to the ‘nurse driven protocol’ (p = 0.017). The additional task for the sonographer to place the peripheral intravenous catheter did not significantly increase the time to complete the study.

**Conclusion:**

Allowing sonographers to administer enhancing agent reduced individual echocardiogram study times by approximately 5 min, supporting that a ‘sonographer driven protocol’ is more efficient with potential downstream economic benefits.

## Introduction

The use of enhancing agent improves the accuracy and reproducibility of transthoracic echocardiograms when non-contrasted images are suboptimal. This can be the case in up to 15% of patients with poor endocardial border definition [1]. Several factors may affect the quality of rest echocardiographic imaging such as body habitus, chest deformities, comorbid conditions to include lung disease, as well as the clinical setting [2, 3]. The indications for clinical use of enhancing agent include facilitating qualitative and quantitative evaluation of left ventricular structure and function, identification of abnormal intracardiac masses, visualization of the right ventricle, and enhancement of Doppler signals for valve function [4]. Suboptimal echocardiograms, defined as the inability to visualize at least 2 of the 6 segments in the apical echocardiographic views, can be salvaged to diagnostic quality in 75–90% of patients with the use of enhancing agent [5–8].

Optison was the first perfluorocarbon-containing intravenous contrast agent approved for use. Its original indication was the enhancement of left venticular opacification. DEFINITY is a lipid-coated microbubble that contains perfluoropropane and has two shell components to include a long-chain lipid and an emulsifier. DEFINITY received approval for endocardial border detection [9].

Despite initial concerns, there is a consensus that contrast echocardiography with the use of enhancing agents is safe. The administration of enhancing agents in pulmonary hypertension, right-to-left shunt through a patent foramen ovale, and in the critically ill has been proven in several studies to be safe. Anaphylactic reaction is a rare complication and has been reported approximately 1 in 10,000 patients [9].

Although there is an abundance of information regarding the safety and benefit of enhancing agents with regard to improving image quality and diagnostic accuracy, the use of these agents has been plagued by barriers and misconceptions. A major hindrance to performing contrast studies is having competent individuals in the laboratory to place the peripheral intravenous catheter and inject enhancing agent with ease. The concept of training sonographers to perform these functions, especially in settings where nurses are not readily available, has been complicated by hospital resistance for personnel other than nurses or physicians to execute these tasks [10].

The present study was designed to evaluate the feasibility of a ‘sonographer driven protocol.’ This involved allowing the sonographer to manage the utilization of enhancing agent with the autonomy to decide if clinically appropriate and ultimately proceed with the administration. Additionally, we determined if allowing the sonographer to place the peripheral intravenous catheter significantly increased the time to complete the study.

## Methods

The outpatient echocardiograms from 3 sonographers were retrospectively queried from March 2015 to June 2016 from a single institution echocardiography laboratory at Brooke Army Medical Center. During the time period of the ‘nurse driven protocol,’ 1033 total echocardiograms were collectively performed in the inpatient and outpatient settings with a 10.6% utilization rate of contrast. The number of echocardiograms performed during the time period of the ‘mixed protocol’ was 1001, with a 32.5% utilization rate of contrast. Finally, during the time period of the ‘sonographer driven protocol’ 931 echocardiograms were performed, and 18% of the studies utilized enhancing agent. Time to complete non-contrasted images, contrast-enhanced images, and total study duration were tracked for comparison by reviewing image acquisition times on the digital imaging and communications headers. During the study periods, the sonographers were not made aware of the intent to measure their performance and echocardiogram completion times. The need for enhancing agent was determined by the sonographers based on their judgement regarding visualization of wall segments during the acquisition of non-enhanced images. Stress echocardiograms, studies utilizing agitated saline, and other adjuncts performed to include 3D echo or strain imaging were excluded for standardization. The enhancing agent used, body mass index (BMI) of the patient, presence of greater than mild valve disease, and the indication or diagnosis for the study were tracked (Table 1).

**Table1:** Baseline characteristics of the study population, enhancing agent, and indication for the echocardiogram across the three protocols by percentage and by mean with standard deviation

	Nurse driven	Mixed protocol	Sonographer driven	p values
*n*	47	85	100	
Enhancing agent (Optison vs. DEFINITY)	26 (55%)	51 (59%)	84 (84%)	< 0.001*
BMI (kg/m^2^)	31.8 ± 4.6	32.4 ± 7.4	30.9 ± 5.9	0.256
Time to complete contrast images (min)	14.4 ± 6.6	10.8 ± 7.8	11.4 ± 7.8	0.116
> Mild valve disease	4 (9%)	4 (5%)	13 (13%)	0.133
Arrhythmia/abnormal electrocardiogram	3 (6%)	7 (8%)	17 (17%)	0.081
Atherosclerotic cardiovascular disease	3 (6%)	7 (8%)	4 (4%)	0.475
Clinical finding	24 (51%)	32 (38%)	40 (40%)	0.307
Chest pain/dyspnea	10 (21%)	19 (22%)	19 (19%)	0.849
Edema	2 (4%)	1 (1%)	2 (2%)	0.538
Heart failure/cardiomyopathy	1 (2%)	2 (2%)	4 (4%)	0.748
Palpitations	2 (4%)	4 (5%)	5 (5%)	0.980
Syncope	0 (0%)	3 (4%)	2 (2%)	0.259
Valvular heart disease	2 (4%)	10 (12%)	7 (7%)	0.267

*indicates a significant trend towards the use of Optison by the sonographers across all three protocols

### Nurse driven protocol

Baseline completion times were obtained from March to June 2015 under the current institutional policy of a ‘nurse driven protocol’ in which a nurse placed the peripheral intravenous catheter and administered enhancing agent after the sonographer determined that contrast enhancement was clinically necessary. Sonographers were instructed to identify patients needing enhancing agent early by either reviewing previous studies or assessing the need at the beginning of the study, and informing the nurse in advance if enhancing agent was going to be utilized. The decision to use enhancing agent was made by the sonographer in real-time with a standing order to utilize if two adjacent segments were not well visualized on apical images, or if the clinical indication was hypertrophic cardiomyopathy or ventricular thrombus in accordance with established ASE guidance. No physician input was required. Sonographers would communicate directly with a nurse to have the enhancing agent administered.

### Mixed protocol

Sonographers received a one-day training on the indications, contraindications, preparation, administration, and monitoring of the available enhancing agents at the institution (Optison or DEFINITY). Training was completed in one business day consisting of live didactics with hands-on demonstration of the proper care and use of a peripheral intravenous catheter. This was followed by a 3 month familiarization period. From September to December 2015, study completion times were obtained for the ‘mixed protocol’ in which a nurse placed the peripheral intravenous catheter and enhancing agent was administered by a trained sonographer.

### Sonographer driven protocol

The sonographers received a one-day training on the placement of a peripheral intravenous catheter and competency was assessed by nurse observation of 5 that were properly placed. This was again followed by a 3 month familiarization period. Study completion times were obtained as part of the ‘sonographer driven protocol’ from March to June 2016, with sonographers placing the peripheral intravenous catheter and administering enhancing agent independently.

### Wall motion

Each echocardiogram included in the study over the 15 month period was analyzed by the same interpreter for wall motion analysis. Visualized wall segments and wall motion were first evaluated in the non-contrasted images. The segments were numbered 1–16 and the presence of wall motion abnormalities binary as either yes or no. The same analysis was performed on the contrast-enhanced images, determining the number of visualized segments and the presence or absence of wall motion abnormalities. Additionally, it was noted whether or not the use of contrast by the sonographer was an appropriate decision. During the review of the non-contrasted images by the interpreter, if there were at least 2 wall segments not well visualized then administration of enhancing agent was considered to be appropriate.

### Statistical analysis

Categorical Data was summarized using percentages and analyzed using Chi-Squared tests. Means and standard deviations were used as summary statistics for continuous variables and were analyzed using ANOVA with a Tukey adjustment for post-hoc pairwise comparisons. Significance for results was established when p values were less than 0.05. All statistical analysis was performed using JMP v13.2 (SAS Corp).

## Results

### Baseline characteristics

A total of 232 patients in the outpatient setting with technically difficult studies requiring enhancing agent administration for endocardial enhancement comprised the study population. Baseline characteristics are listed in Table 1. There was a statistically significant favor towards the use of Optison (p < 0.001). Otherwise, factors that might lead to increased imaging time such as BMI of the patient population, the diagnosis or indication for the echocardiogram, and presence of greater than mild valve disease was not statistically significant across the three protocols. The indication or diagnosis of ‘clinical finding’ derived from the consult for the study was the most commonly utilized in each of the protocols. Time to complete only the contrast-enhanced images was not significantly different between the protocols or sonographers (p = 0.116). Appropriate use of enhancing agent by the sonographer was greater than 97% (226 of 232 studies). No adverse events were observed in any study period from either administration of enhancing agent or peripheral intravenous catheter.

### Impact of enhancing agent administration by sonographer

**Fig. 1:**
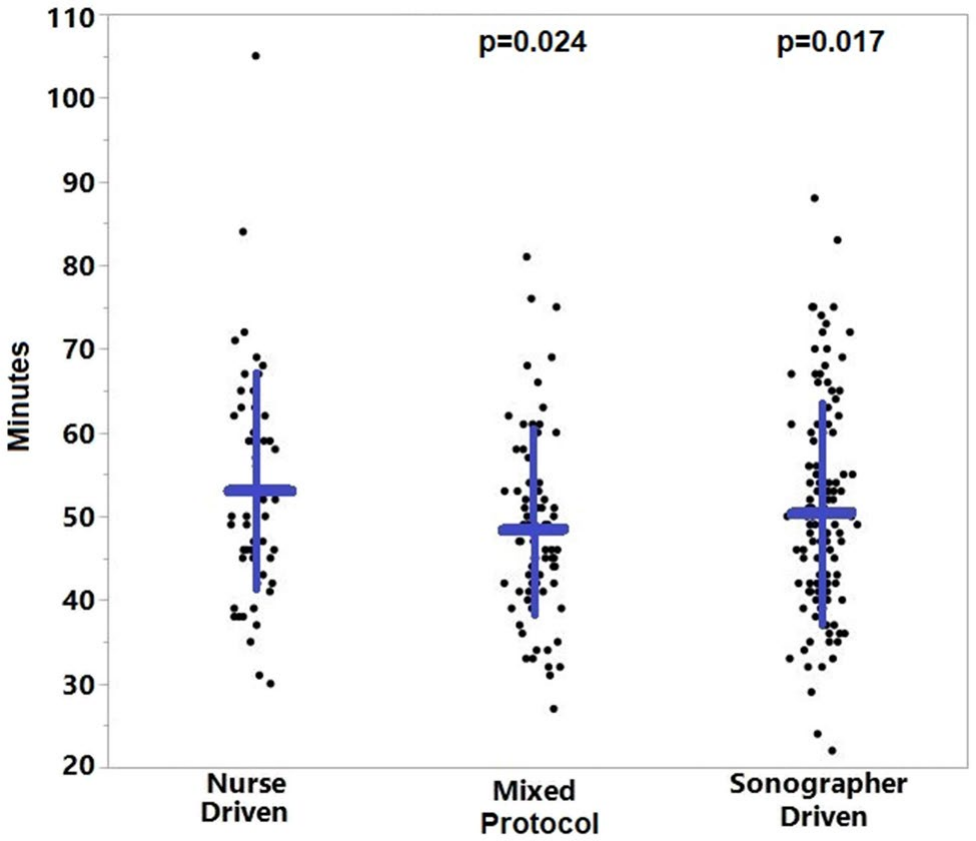
Comparison of echocardiogram study completion times across the three protocols. p values listed are compared to the Nurse Driven protocol. The blue horizontal lines represent the mean and the vertical lines represent one standard deviation

When comparing the ‘mixed protocol’ (49.4 min ± 11.4) and the ‘nurse driven protocol’ (54.6 min ± 12.9), there was a significant difference in time spent to complete each echocardiogram with a p = 0.024. This analysis favored the administration of enhancing agent by the sonographer in the ‘mixed protocol’ as demonstrated in Fig. 1 with a reduction in time to complete the exam.

### Impact of peripheral intravenous catheter placement and enhancing agent administration by sonographer

The time required to complete each exam was less with the ‘sonographer driven protocol’ (50.3 min ± 12.6) in comparison to the ‘nurse driven protocol’ (p = 0.017). This indicates that requiring the sonographer to place a peripheral intravenous catheter in addition to administering enhancing agent did not significantly increase the time required to complete the exam. Analysis demonstrated a significant reduction in time to complete the exam in comparison to utilizing nursing personnel (Fig. 1).

## Discussion

Our institutional study demonstrated that the implementation of a ‘sonographer driven protocol’ resulted in a reduction in time to complete routine outpatient transthoracic echocardiograms that had suboptimal endocardial border definition requiring the use of enhancing agent. Empowering the sonographer to also place the peripheral intravenous catheter resulted in time saved in comparison to the protocol requiring nursing personnel. Additionally, the sonographers were able to correctly identify the need for enhancing agent 97% of the time. Time saved between the latter two protocols was secondary to the sonographer not needing to wait for nursing personnel to come and assist in the administration of the enhancing agent. The time saved, accumulating to approximately 30 min a day depending on the number of scheduled scans, may potentially allow for an additional scan to be performed each day. Eliminating the need for nursing staff to administer enhancing agent and/or place the peripheral intravenous catheter also results in cost savings, as the average hourly salary of a registered nurse employed in Texas is $25–$48 depending on level of experience [11]. Limitation or exclusion of nursing personnel did not negatively affect the utilization rate of enhancing agent, and instead, promoted its use. This is evident by the increase in the amount of studies overall with contrast images across the three protocols (from 10.8 to 32.5%). Not only was there a trend towards increased use of enhancing agent, but our study also demonstrates that sonographer autonomy resulted in appropriate use. Sonographer preference powered the use of Optison over DEFINITY.

There are several described complications of intravenous therapy to include phlebitis, thrombophlebitis, infiltration, extravasation, and infection. Competency of the personnel placing the catheter influences the incidence of these complications. This drove the training initiatives and expectations implemented for our sonographers, to include direct observation of at least the first five peripheral intravenous catheters placed to ensure competency. Additionally, the ability to obtain venous access on the initial attempt can have an impact on health care costs, and the number of attempts to place a peripheral intravenous catheter influences labor and supply costs. [12].

**Fig. 2:**
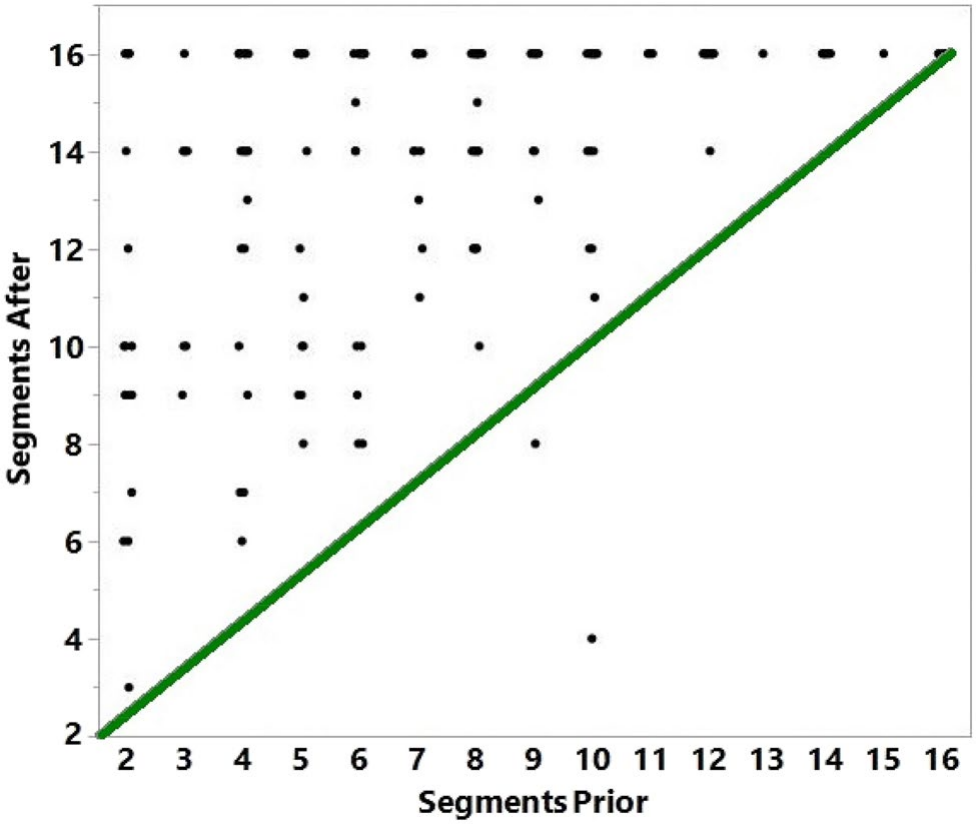
Comparison of wall segments visualized prior to contrast administration versus after. The green line represents the line of identity

With the advent of contrast echocardiography, the percentage of nondiagnostic echocardiography studies has dropped to less than 5% [13]. Our statistical analysis demonstrated an improvement in visualization of left ventricular segments after contrast use in comparison to non-contrasted images (Fig. 2). This is to be expected as improved endocardial border definition with contrast-enhanced echocardiography has been previously shown to result in better reader confidence for interpreting wall motion [7]. Hundley et al., demonstrated in those patients with poor endocardial border definition that the use of enhancing agent improved visualization of the endocardium and assessment of regional wall motion, and that the ability to delineate normal versus abnormal left ventricular wall thickening with enhanced echocardiography was similar to that of cine MRI [14]. The use of enhancing agent has also allowed for better reproducibility and less intraobserver and interobserver variability in regard to measured left ventricular volumes and ejection fraction [15]. Quantification of ejection fraction is imperative in daily practice both in clinical decision-making and in performing serial studies [16].

A limitation of our study is that while we observed that our sonographers were able to appropriately identify the need for an enhancing agent 97% of the time based on our review of the performed contrasted studies, we did not review the non-enhanced studies to evaluate any requiring enhancing agent that were missed. Additionally, during the time period of the reviewed echocardiograms our institution was not tracking inpatient versus outpatient echocardiograms separately, therefore, we are only able to provide the amount of total echocardiograms performed during these specified intervals.

Appropriate and timely use of enhancing agent requires a team approach involving sonographers, nurses, and physicians. Castello, et al., determined through their institutional research that allowing the sonographer to determine the need for contrast for left ventricular assessment was clinically effective and efficient [17]. This finding is mirrored in our study. Given the clear benefit that contrast-enhanced echocardiography results in increased diagnostic accuracy, it is imperative that there is a streamlined policy implemented to maximize its use [7, 14, 15, 18]. If this is achieved, the quantity of patients to benefit from the use of enhancing agent and more diagnostically accurate echocardiograms can be significantly increased. Additionally, as reflected in our research, reducing the completion time of each study requiring enhancing agent by at least 5 min could lead to the allotment of additional studies to be performed in a given work day, and expected to result in cost savings with less need for nursing personnel.
